# Prognostic implications of calculated Apo‐lipoprotein B in patients with ST‐segment elevation myocardial infarction undergoing primary percutaneous coronary intervention: Outcome is tied to lower cut‐points

**DOI:** 10.1002/clc.23610

**Published:** 2021-05-04

**Authors:** Saeed Ghodsi, Mehrnaz Mohebi, Seyed‐Ali Sadre‐Bafghi, Hamidreza Poorhosseini, Mojtaba Salarifar, Mohammad Alidoosti, Ali‐Mohammad Haji‐Zeinali, Alireza Amirzadegan, Hassan Aghajani, Yaser Jenab, Zahra Hosseini

**Affiliations:** ^1^ Research department, Tehran Heart Center Tehran University of Medical Sciences Tehran Iran; ^2^ Department of Cardiology, Tehran Heart Center Tehran University of Medical Sciences Tehran Iran; ^3^ Research center at department of Cardiology Imam Khomeini Hospital Complex, Tehran University of Medical Sciences Tehran Iran

**Keywords:** Apo‐B, lipoproteins, MACE, primary PCI, revascularization, STEMI, survival

## Abstract

**Background:**

Debates still surround using lipoproteins including Apo‐B in risk assessment, management, and prognosis of patients with coronary artery disease. During an acute ST‐segment elevation myocardial infarction, Apo‐B might help to achieve incremental prognostic information.

**Objective:**

We sought to determine the potential prognostic utility of calculated Apo‐B in a cohort of patients with STEMI undergoing primary PCI.

**Methods:**

A retrospective cohort study was conducted enrolling 2,259 patients with a diagnosis of acute STEMI who underwent primary PCI. Apo‐B was obtained using a valid equation based on initial lipid measurements. High Apo‐B was defined as a level of 65 or higher. Primary endpoint of the study was major adverse cardiovascular events (MACE).

**Results:**

Mean age of the participants was 59.54 years and 77.9% of them were male. After a Median follow up of 15 (6.2) months, high Apo‐B was associated with MACE and the OR (95% CI) was 3.02 (1.07–8.47), p = .036. Odds ratios for prediction of MACE pertaining to LVEF, and smoking were 0.97 (p = .044), and 1.07 (p = .033), respectively. However, High Apo‐B was not able to predict suboptimal TIMI flow. Accordingly, the Odds ratio was 0.56 (0.17–1.87), p = 0.349. The power of High LDL‐C and Non‐HDLC for prediction of MACE were assessed in distinct models. Attained odds ratios were [2.40 (0.90–6.36), p = .077] and [1.80 (0.75–4.35), p = 0.191], respectively.

**Conclusion:**

Calculated Apo‐B appears to be a simple tool applicable for prediction of cardiovascular events in patients with STEMI superior to both Non‐HDLC and LDL‐C.

**Abbreviations**
ACC/AHA: American college of cardiology/American Heart Association
ACEI/ARB: angiotensin converting enzyme inhibitor/ angiotensin receptor blocker
Apo‐B: Apo lipoprotein‐B
BMI: body mass index
CABG: coronary artery bypass graft
CAD: coronary artery disease
EAS: European atherosclerosis society
ESC: European society of cardiology
ESRD: end stage renal disease
HDL: high‐density lipoprotein
IDL: intermediate‐density lipoprotein
LAD: left anterior descending artery
LCX: left circumflex artery
LDL‐C: low‐density lipoprotein
LVEF: left ventricle ejection fraction
MACE: major adverse cardiovascular events
MI: myocardial infarction
Non‐HDLC: non‐high density lipoprotein cholesterol
OR: odds ratio
PCI: percutaneous coronary intervention
RCA: right coronary artery
SIHD: stable ischemic heart disease
STEMI: ST‐segment elevation myocardial infarction
TG: triglyceride
TIMI: thrombolysis in myocardial infarction
VLDL‐C: very low density lipoprotein

## 1 INTRODUCTION

Timely primary PCI that restores coronary perfusion is the mainstay of STEMI management.^1^ Subsequent cardiovascular events are mainly affected via risk factor modification as well as medical treatment. Thus, we need instruments such as lipid profile in order to assess the cardiovascular risk and to guide medical treatment. Recent epidemiologic and clinical studies have highlighted the role of measured Apo‐lipoprotein B 100 (Apo‐B) as a powerful predictor of ischemic events superior to low‐density lipoprotein (LDL‐C) cholesterol.^2^, ^3^ However, calculation of Apo‐B with an equation appears more feasible than direct measurement providing a relatively simple tool for risk stratification.

Lipoprotein fractions such as VLDL‐C, IDL, and LDL‐C are comprised of structural proteins like Apo‐B. These pro‐atherogenic lipid particles contain one Apo‐B component. Apo‐B that is synthesized in the liver facilitates and amplifies cholesterol transfer in a cycle streaming from the liver to peripheral tissues.^4^ Hence, precise estimation of serum Apo‐B concentration indicates to burden of atherogenic lipoprotein particles.^5^ Despite the utility of Apo‐B in prediction of future cardiovascular events among general population and patients with stable CAD,^6^, ^7^ its clinical implications in STEMI remains to be determined. Measured Apo‐B has shown incremental prognostic value in post‐MI patients in few clinical studies which was superior to both LDL‐C and Non‐HDLC.^8^, ^9^Nevertheless, Calculation of Apo‐B has not been validated to enter the routine practice, particularly in the setting of STEMI. Furthermore, multiple large‐scale studies are required to confirm the clinical relevance of an equation. In the present research, we aimed to investigate potential association of calculated Apo‐B with cardiovascular events following primary PCI in high‐risk STEMI patients. Furthermore, we assessed the power of high LDL‐C and Non‐HDLC in prediction of major adverse cardiovascular events (MACE).

## 2 METHODS

### 2.1 Study design and participants

We performed a thorough review of Tehran Heart Center (THC) registry for CAD. Patients with the diagnosis of STEMI (ST‐elevation myocardial infarction) since March 2016 up to Juli 2019 were enrolled. We arranged a retrospective cohort design. All individuals with acute STEMI who underwent primary PCI or rescue PCI within 24 h since the onset of symptoms were included. Subjects in whom the initial presentation of MI was unclear or those with failed wire passage were excluded. All demographic characteristics, biochemical parameters, variables of clinical history, and physical examination were obtained from the database. Baseline Left ventricular Ejection fractions (LVEF) were used in the current study, which were determined at least 7 days post‐MI. Cardiac Troponin‐T was measured at baseline and 12 and 24 h following primary PCI denoting the extent of myocardial damage. In the current study, we applied the maximum level of Troponin‐T in analyses.

All patients received the standard optimal guideline‐directed medical treatment in combination with angioplasty. Either an ACEI or ARB agent, an appropriate beta‐blocker, Aspirin (80 mg daily) and high‐dose statin were administered for all participants. Aldosterone receptor antagonist had been prescribed for those with an LVEF under 40% or diabetics. All patients received a P2Y12 inhibitor drug including clopidogrel and ticagrelor. Loading dose of clopidogrel and ticagrelor were 600 mg and 180 mg, respectively. Then maintenance dose of the former (75 mg once daily) and the latter (90 mg twice a day) were continued for at least 12 months, thereafter. Unfractionated heparin (70‐to 100‐unit /kg) was used routinely during primary PCI. The choice of using thrombus aspiration and/ or glycoprotein IIb‐IIIa inhibitors were optional depending on the opinion of the interventional cardiologist.

### 2.2 Definitions

STEMI was diagnosed according to the fourth universal definition of myocardial infarction.^10^In brief, ischemic symptoms such as chest pain or its equivalents accompanied with ECG changes in two or more contiguous leads led to definite diagnosis. ST‐segment elevation of 1 mm or more in at least two related leads except for V2 and V3 fulfills the criteria. In these two leads, a value of 2 mm or more is needed to confirm STEMI for men older than 40 years. In younger men (under 40) the cut‐point of ST elevation in V2 and V3 is 2.5 mm while for women at any age, this measure is expected to reach 15 mm or more.

TIMI flow grade is a surrogate measure of myocardial perfusion, which is detected via inspection of angiography movies. Operators had recorded TIMI flow of the coronary arteries before and after PCI. This visual classification also helps to determine apparent success of the procedure.^11^ Absence of any forward coronary flow is reported as TIMI grade zero whereas TIMI‐1 addresses minimal presence of dye just beyond the stenosis. TIMI‐2 characterizes a delayed slow filling of the distal segment in the culprit territory. Optimal antegrade stream is known as TIMI‐3.

Estimated Apo‐B was derived from an equation using serum concentrations of LDL‐C and natural logarithm of triglyceride (ApoB = −33.12 + 0.675*LDL‐C + 11.95*ln [TG]). Calculated Apo‐B with mentioned equation was first validated in a large‐scale Asian population with 73 047 participants. This formula appears to have an ample power in prediction of measured Apo‐B levels with a concordance correlation coefficient (95% CIs) = 0.936 (0.935–0.937).^12^ We used the target of Apo‐B in very‐high risk patients as the cut‐off for determining high level according to 2019 ESC/EAS Guidelines for the management of dyslipidemias.^13^ Thus, Apo‐B values above 65 were defined as high. Elevated LDL‐C and Non‐HDLC were determined by levels ≥ 70, and ≥ 100, respectively.

### 2.3 Endpoints

Primary endpoint of the study was Major Averse Cardiovascular Events (MACE), which was a composite of all‐cause mortality, repeat revascularization by PCI or CABG, and non‐fatal MI. Secondary endpoints were suboptimal TIMI flow as well as components of MACE. Routine follow‐up of the patients were performed by regular (5 months intervals post‐PCI) visits at THC clinics or phone calls at predetermined intervals.

Ethics committee of Tehran University of Medical Sciences has approved the protocols of data collection in THC registry as well as the current study. Principles of declaration of Helsinki were also met in this research.

### 2.4 Statistical analysis

Continuous variables were shown as mean ± *SD* or median (Interquartile range) with respect to presence or absence of normal distribution. Categorical variables were reported by percentage. Kolmogorov–Smirnov Test and Shapiro–Wilk test were applied to determine the normality of distributions. We used *t* test and Mann–Whitney U test to compare the differences of continuous variables between two groups with and without normal distribution, respectively. Chi‐square test was used for comparison of categorical variables. Statistical significance was achieved for p values under .05 for all tests. All statistical analyses were performed via SPSS version 22.0 (SPSS Inc, Chicago, IL). We have displayed multivariable associations between Apo‐B and MACE. Multivariate regression analysis was also performed to evaluate the predictors of suboptimal post‐PCI TIMI flow. Kaplan–Meier graphs were recruited to illustrate the event‐free survival of Apo‐B subgroups.

## 3 RESULTS

A total of 2259 participants were eligible for analysis in this study. Mean age of the study population was 59.54 ± 11.83 years and majority of the patients were male (77.9%). Mean LDL‐C and calculated Apo‐B levels were 103.82 ± 34.58 and 94.41 ± 25.32, respectively. Average of the Non‐HDLC was 120.43 ± 37.91. Mean LVEF of the population was 42.13 ± 9.2. The average time since early symptoms to arrival known as pain‐to‐door time was 545.26 ± 72.7 min. Statins and Aspirin were initiated and continued during follow‐up period in almost all patients. Table 1 represents the baseline characteristics of the two groups with low and high Apo‐B. Patients in end stage renal disease (ESRD) who required regular hemodialysis comprised 1.0% (6) of low Apo‐B and 0.1% (2) of high Apo‐B categories, respectively (p = .042). The frequency of prior CABG and PCI were greater among patients with low Apo‐B group compared with that of high Apo‐B group. Corresponding values pertaining to previous CABG and PCI were (8.7% vs 3.7%, p = .0011) and (16.4% vs 9.4%, p = .016), respectively. History of stroke was reported in 5.8% (34) and 3.5% (58) of patients with low and high Apo‐B, respectively. Calculated p value was 0.14 in this comparison. Median (interquartile range) of follow up time was 15 (6.2) months.

**TABLE 1:** Baseline characteristics of the patients with STEMI undergoing primary PCI

	Low Apo‐B (N = 586)	High Apo‐B (N = 1673)	p value
Age	62.84 ± 13.13	58.21 ± 11.90	.066
Sex (Male)	76.1% (446)	78.5% (1313)	0.234
BMI	26.79 ± 4.70	27.70 ± 3.77	0.361
LVEF% Median (IQR)*	40 (15)	45 (10)	.054
DM	40.3% (236)	38.4% (642)	0.417
Tobacco smoking			
Current	29.9% (175)	36.5% (611)	.002
Former	11.3% (66)	7.8% (131)	.019
Opium use			
Current	10.8% (63)	10.8% (181)	0.964
Former	5.3% (31)	4.5% (76)	0.557
PCI location			
Ostial	14.7% (86)	11.6% (194)	.027
Proximal	36.5% (214)	33.6% (562)	.021
Mid/distal	48.8% (286)	54.8% (9'7)	.007
Hemoglobin	14.41 ± 2.05	15.12 ± 1.87	0.128
Creatinine *	0.90 (0.32)	0.90 (0.30)	.058
FBS*	108 (42)	113 (61.75)	.071
Troponin‐T (max) *	2573 (1825)	2404 (1789)	0.951
HDL	38.37 ± 14.34	38.14 ± 9.83	0.461
Lesion length (mm)*	25 (18)	24 (16)	0.387
Hypertension	52.9% (310)	41.4% (692)	.041
Optimal final TIMI (3)	21.7% (127)	19.4% (325)	0.328
GpIIb‐IIIa use	65.2% (382)	65.9% (1117)	0.767
Cardiogenic shock	1.0% (6)	1.2% (20)	0.738
Pain‐to‐door time (min)	593.37 ± 44.16	595.97 ± 25.64	.074
MI (PPCI) territory			
LAD	49.1% (288)	48.7% (814)	0.425
LCX	16.7% (98)	18.6% (312)	0.281
RCA	29.4% (172)	30.4% (509)	0.316
SVG	4.8% (28)	2.3% (38)	.023

Abbreviations: BMI: body mass index; DM, diabetes mellitus; FBS, fasting blood sugar; LVEF, left ventricle ejection fraction; PPCI, primary PCI; PCI, percutaneous coronary intervention, TIMI, thrombolysis in myocardial infarction, GpII‐IIIa, glycoprotein IIb‐IIIa, LAD, left anterior descending artery; LXC, left circumflex artery; RCA, right coronary artery, SVG: saphenous vein graft.

*Note*: Continuous variables are expressed as Mean ± *SD* or Median* (Interquartile range) while categorical variables are shown using percentage (count).

The association of Apo‐B as a continuous variable with MACE was determined via multivariate regression. The Odds ratio adjusted for age, sex, hypertension, LVEF, and TIMI flow was 1.002 (1.001–1.007), p = .047 per 10 units increment of Apo‐B. We categorized the estimated Apo‐B into four equal groups, Thereafter. Highest versus the lowest quartile (Q4 / Q1) was related to MACE with an Odds ratio of 1.12 (1.08–1.36). However, third versus first quartile (Q3 / Q1) as well as Q2/Q1 did not show a significant relationship with MACE. Corresponding Odds ratios were 1.16 (0.77–1.74) and 0.65 (0.41–1.03), respectively. These relations were also adjusted for age, sex, hypertension, LVEF, and TIMI flow.

Table 2 shows the association of dichotomized Apo‐B and subsequent MACE in patients following primary PCI in multivariate models. In Table 3 potential relation of high LDL‐C and primary endpoint has been evaluated. High Apo‐B predicted greater incidence of MACE while the association between high LDL‐C and MACE was not statistically significant. Odds ratios (95% CI) pertaining to high Apo‐B and high LDL‐C were: (OR = 3.02 [1.07–8.47], p = .036) and (OR = 2.40 [0.90–6.36], p = .077), respectively.

**TABLE 2:** Multiple logistic regression models pertaining to high Apo‐B and other predictors of MACE in patients with STEMI undergoing primary PCI

		Model 1 Odds Ratio (95% CI)	p value	Model 2 Odds Ratio (95% CI)	p value
High Apo‐B (> 65 vs <65)		3.02 (1.07–8.47)	.036	2.92 (1.13–7.55)	.027
FBS		0.99 (0.98–1.01)	.097	0.99 (0.98–1.01)	.080
BMI		0.909 (0.81–1.02)	0.105	0.929 (0.838–1.031)	0.166
LVEF(per 5 percent increase)		0.97 (0.86–0.99)	.044	0.96 (0.73–0.96)	.039
DM		1.87 (0.69–5.49)	0.219	1.88 (1.07–8.47)	0.193
Tobacco smoking		1.07 (1.01–3.97)	.033	1.12 (1.03–4.31)	.024
Opium use		1.87 (0.86–4.01)	0.106	1.68 (0.81–3.47)	0.162
PCI location (mid/distal vs proximal/ostial)		0.65 (0.38–1.11)	0.111	0.66 (0.40–1.08)	.091
Cardiogenic shock		3.81 (0.174–82.21)	0.395	6.63 (0.79–89.31)	0.154
Age		1.01 (0.98–1.05)	0.415		
Post‐PCI TIMI	Slow flow (2) vs (0,1)	2.86 (0.24–33.77)	0.404		
	Normal flow (3) vs (0,1)	2.12 (0.42–10.67)	0.363		
Hemoglobin		1.15 (0.89–1.48)	0.294		
Sex		0.44 (0.135–1.45)	0.176		
Hypertension		1.15 (0.47–2.08)	0.766		
GpIIb‐IIIa use		1.38 (0.51–3.73)	0.521		

*Note*: Model 1: The associations were adjusted for serum creatinine, HDL, Troponin‐T(maximum level), Coronary Lesion length, prior stroke, prior angioplasty, Prior CABG, territory of MI, and Pain‐to‐door time. Model 2 provides a relatively reduced scheme than model 1.

Abbreviations: BMI, body mass index; DM, diabetes mellitus; FBS, fasting blood sugar; LVEF, left ventricle ejection fraction; PCI, percutaneous coronary intervention; TIMI, thrombolysis in myocardial infarction; GpII‐IIIa, glycoprotein IIb‐IIIa.

**TABLE 3:** Multiple logistic regression showing the role of high LDL in predicting MACE in patients with STEMI

Predictors		Odds ratio (95% CI)	p value
High LDL (> 70 vs <70)		2.40 (0.90–6.36)	.077
FBS		0.99 (0.97–1.02)	.082
BMI		0.90 (0.79–1.02)	0.103
LVEF(per 5 percent increase)		0.95 (0.90–0.98)	.048
DM		1.48 (0.51–4.22)	0.465
Tobacco smoking		1.11 (1.02–3.18)	.045
Opium use		2.06 (0.96–4.44)	.065
PCI location (mid‐distal vs proximal/ostial)		0.65 (0.38–1.14)	0.131
TG (per 1 unit rise)		1.004 (1.001–1.008)	.012
Age (per 1 years)		1.01 (0.98–1.05)	0.415
Post‐PCI TIMI	Normal (3 vs < 3)	0.47 (0.16–1.41)	0.178

*Note*: The associations were adjusted for sex, HDL, Coronary Lesion length, Troponin‐T(maximum level), COPD, Creatinine, Hemodialysis. Territory of MI, Cardiogenic shock, Hemoglobin, prior stroke, Prior angioplasty, Prior CABG, Pain‐to‐door time, Hypertension, and GpII‐IIIa use.

Abbreviations: BMI, body mass index; CABG, coronary artery bypass graft; COPD, chronic obstructive pulmonary disease; DM, diabetes mellitus; FBS, fasting blood sugar; LVEF, left ventricle ejection fraction; TG, triglyceride PCI, percutaneous coronary intervention; TIMI, thrombolysis in myocardial infarction.

Non‐HDLC level greater than 100 mg/dl was not significantly associated with MACE. Multivariate logistic regression demonstrated that odds ratio of elevated Non‐HDLC was 1.80 (0.75–4.35), p = 0.191. Co‐variates in this regression analysis and related adjustments were performed in the same way as those in models for Apo‐B and high LDL‐C.

Table 4 pertains to multivariate analysis demonstrating the association of ApoB and final Suboptimal TIMI flow (<3) in patients with STEMI.

**TABLE 4:** Multiple logistic regression demonstrating the association of ApoB and final Suboptimal TIMI flow (<3) in patients with STEMI diagnosis undergoing primary PCI

	Odds Ratio (95% CI)	p value
High Apo‐B (> 65 vs <65)	0.56 (0.17–1.87)	0.349
Creatinine (per 0.2 mg/dl)	1.59(1.09–2.31)	.015
FBS	0.996 (0.990–1.013)	0.176
BMI	1.06 (0.95–1.18)	0.263
LVEF(per 5 percent increase)	0.928 (0.885–0.974)	.002
Cardiogenic shock	25.93 (3.87–53.21)	.003
Pre‐PCI TIMI (>2 vs ≤2)	0.587 (0.342–0.98)	.045
PCI location (non‐proximal vs proximal/ostial)	1.34 (0.71–2.50)	0.368

*Note*: The associations were adjusted for age, sex, HDL, Troponin‐T(maximum level), Territory of MI, Pain‐to‐door time, HTN, and GpII‐IIIa use.

Abbreviations: BMI, body mass index; FBS, fasting blood sugar; LVEF, left ventricle ejection fraction; PCI, percutaneous coronary intervention; TIMI, thrombolysis in myocardial infarction.

**FIGURE 1:**
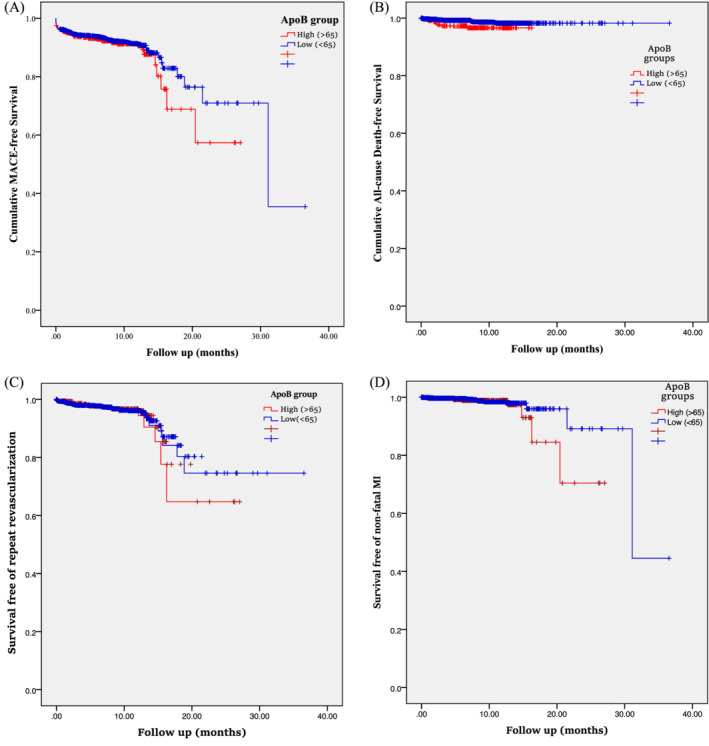
MACE‐free survival of STEMI patients with high and low Apo‐B levels. B, Mortality‐free survival of STEMI patients with high and low Apo‐B levels. C, Survival free of repeat revascularization in STEMI patients with and without high Apo‐B undergoing primary PCI. D, Survival free of non‐fatal MI in STEMI patients with and without high Apo‐B undergoing primary PCI

Kaplan–Meier graphs also depicted event‐free survivals comparing high‐ and low Apo‐B groups (Figure 1–(D)). A subgroup analysis was also performed to demonstrate the differences of main effects regarding the association of dichotomized Apo‐B and MACE. Overall, a significant relationship or a trend toward significance appeared in majority of the subgroups. Figure S1 (in supplementary file) illustrates a forest plot addressing to the mentioned subgroup analysis.

## 4 DISCUSSION

To date, Apo‐B 100 measurement has improved cardiovascular risk stratification in various conditions including acute coronary syndrome (ACS). However, Apo‐B assays are often costly and not readily available particularly in acute settings such as STEMI. Given this challenge, we recruited a previously introduced simple equation to calculate Apo‐B. Analyses demonstrated that increased baseline Apo‐B concentrations could predict the outcome of patients after primary PCI. On the contrary, the association of high LDL‐C and MACE was not confirmed even though a trend toward significance was observed. Besides, Non‐HDLC also did not show the ability to predict the outcomes. As the main finding, results suggest that calculated Apo‐B might have greater prognostic benefit than measured LDL‐C and Non‐HDLC after STEMI.

There are few studies investigating the role of Apo‐B in early phase post‐STEMI. However relatively similar articles have been discussed here. In contrast with our results, a large‐scale cohort of ACS patients found no significant relationship between high Apo‐B and MACE over 12 months.^14^ They have also reported an inverse association between Apo‐B and adverse outcomes in a subgroup of women. However, their subjects were younger (10 years in average) and a small proportion of their participants were adhered to statin treatment. On the other hand, there are more studies almost in concordance with our findings.^7^, ^8^, ^9^, ^15^, ^16^, ^17^, ^18^, ^19^, ^20^ These studies are very heterogeneous due to different clinical contexts, population enrolled, timing of lipoprotein assays, and underlying treatment options. Most of these researches have been performed among low‐risk individuals or patients with stable ischemic heart disease (SIHD) while our study is confined to STEMI. In this regard, two large studies named INTERHEART^8^ and IDEAL^9^ had enrolled Post‐MI patients. These two papers underscore the superiority of Apo‐B levels to LDL‐C and Non‐HDLC in prediction of cardiovascular outcomes. Likewise, few reports among patient populations other than ACS including asymptomatic subjects,^3^, ^21^, ^22^ SIHD^16^, ^23^ or diabetics,^24^ demonstrated similar findings. We have also found the same results regarding the utility of Apo‐B in patients with STEMI following primary PCI.

Since measurement of Apo‐B is time consuming and not available as a routine test, we recruited an equation, which serves as a simple surrogate of this biomarker. Using only one baseline blood sample and the patient's conventional lipid profile, we are able to determine a modestly strong predictor of MACE in acute STEMI. We applied different cut‐off levels of Apo‐B to achieve considerable clinical and statistical significance because it was the first time for validation of prognostic utility of calculated Apo‐B in STEMI. Ultimately, we found that the lowest level (65 mg/dl), which is, defined as the goal of therapy in very high‐risk patients^13^ like those with an atherosclerotic coronary event might render the best result. Hence, the interesting point in the current research is the use of a former Apo‐B therapeutic target as a new prognostic cut‐point at initial visit just before primary PCI. This might reflect the difficulty in assessment of residual risk during an already high‐risk event such as STEMI. Thus, selection of an appropriate Apo‐B level for further baseline risk stratification seems valuable.

The potential privilege of Apo‐B over both LDL‐C and Non‐HDLC origins from the concept of mismatch between LDL‐C concentration and LDL‐C particle number. A meta‐analysis of 11 clinical trials of statin treatment among 17 035 patients illustrated a substantial discrepancy between gained population percentiles of LDL‐C, Non‐HDLC and Apo‐B targets.^25^ The analysis showed greater reductions in LDL‐C (42.1%) and Non‐HDLC (39.6%) rather than that of Apo‐B (33.1%) in response to lipid lowering treatments. Thus, LDL‐C, Non‐HLD and Apo‐B reached to the 21st, the 29th, and the 55th percentiles, respectively. Although in this meta‐analysis, the average of achieved LDL‐C (99.2 mg/dl) and Apo‐B (101.6 mg/dl) were nearly close, but corresponding population percentiles were far away from each other. In fact, receiving a course of statins changes the correlation of Apo‐B and LDL‐C levels. Given this finding, we used only baseline Apo‐B derived from initial serum LDL‐C and TG concentrations. Furthermore, a patient might be at increased risk of future MACE due to high Apo‐B despite achieving an optimal LDL‐C level. Hence considering tighter Apo‐B control (lower cut‐points like 65 mg/dL), may lead to better outcomes.

A recent case–control study with 10‐year follow‐up revealed that increased Apo‐B concentrations (> 100 mg/dl) were associated with first STEMI in asymptomatic controls. However, they declared that neither Apo‐B nor other plasma lipids did not predict MACE in patients with STEMI. These results are relatively in discordance with our findings.^26^ Therefore, we should note that length of follow‐up as well as Apo‐B cut‐off level influence on the subsequent risk. Thus the effects of baseline Apo lipoproteins on magnitude of the residual risk blunts over a long period. Only one study in a diabetic population has applied the cut‐point of 63 mg/dl that is similar to our level.^27^ They found that high Apo‐B was associated with MACE and particularly subsequent non‐fatal MI after index ACS. However, in our study, repeat revascularization was the main cause of difference but non‐fatal MI just showed a trend toward significance.

We found no reports about the correlation of high Apo‐B and sub‐optimal reperfusion, which is in agreement with the present analysis.

The stability, synthesis, and transportation of lipoproteins are regulated via various Apo lipoprotein components. Atherogenic lipoproteins such as very low‐density lipoproteins (VLDL), intermediate‐density lipoproteins (IDL), lipoprotein (a), and LDL‐C contain one molecule of Apo‐B. Thus, serum Apo‐B represents for total number of atherogenic particles and TG‐rich lipoprotein remnants. The higher serum Apo‐B concentration, the greater circulating cholesterol content and ultimately higher risk of atherosclerotic plaque deposition.^28^ Variations in size of LDL‐C particles accompanied with greater atherogenic capacity of small dense LDL‐C as well as TG‐rich remnants should also be addressed. With these in mind, using Apo‐B prevents underestimation of cardiovascular risk.^29^ Moreover, the calculated Apo‐B incorporates TG with logarithmic scale in order to modify the impact of extreme values particularly those over 400 mg/dl. In the present study, it was also depicted that a strong relation exists between increased TG levels and subsequent MACE. This might signify the controversial role of TG‐rich particles and turn the lights on the dark side of the scene. Although hypertriglyceridemia is a well‐established risk factor of coronary artery disease and mortality, a paradoxical effect on prognosis of ACS patients have been suggested.^30^ Albeit, these reports often did not differentiate between the subgroups with STEMI and Non‐STEMI. Conversely, a recent large study postulate that the paradoxical correlation of baseline lipid profile with MACE is limited to serum LDL‐C.^31^

Increased TG‐rich lipoproteins, low HDL‐C and usually normal LDL‐C levels characterize lipid profile of subjects with metabolic syndrome and insulin resistance. However, frequency of atherogenic particles including small dense LDL‐C increases that tend to get oxidized.^32^ Hence, Apo‐B might be an appropriate marker to detect and optimize the true residual risk of STEMI patients who have underlying metabolic syndrome. Estimated Apo‐B is thought to be associated with hs‐CRP, microalbuminuria, Agatston calcium score^33^ as well as SYNTAX score.^20^ Furthermore, a robust correlation was found between Apo‐B reduction and regression of atherosclerotic plaques (coronary plaque volume).^34^ Accordingly, Apo‐B serves as a useful biomarker providing incremental risk assessment through linking with multiple potential risk indicators. In addition, it addresses the severity of coronary artery disease, which is likely to occur in the setting of acute coronary syndrome.^17^

### 4.1 Study limitations

Several limitations might be considered in the present research. First, we have performed a retrospective cohort study, which encompasses known inherent biases. Second, phone call follow‐up might potentially affect the reliability of event records. Pain‐to‐door time was relatively prolonged in this cohort, which elevates the underlying risk and diminishes the myocardial salvage for both groups. Third, we did not have repeated measurements of lipid markers in order to determine the efficacy of statin treatment. Fourth, despite finding a significant relationship between high Apo‐B and poor STEMI outcome, we cannot strongly extrapolate the results. This caution should be considered due to several reasons including relatively broad confidence intervals, and lacking patients with other types of CAD.

## 5 CONCLUSION

The present study helped to validate the efficacy of a simple equation for estimation of Apo‐B in patients with STEMI. Clinical implications of calculated Apo‐B was shown as evidenced for measured Apo‐B previously. High Apo‐B predicted subsequent MACE following primary PCI whereas, elevated Non‐HDLC and LDL‐C failed to have a significant association.

## CONFLICT OF INTEREST

There was no relationship with any institution as well as no contracts related to industry.

## AUTHORS' CONTRIBUTIONS

Hamidreza Poorhosseini and Saeed Ghodsi presented the core concept of the research. Alireza Amirzadegan, Yaser Jenab, Hassan Aghajani, Mojtaba Salarifar, Mohammad Alidoosti, Ali‐Mohammad Haji‐Zeinali performed treatment of the patients including primary PCI. Mojtaba Salarifar and Yaser Jenab supervised data entry and accomplishment of the study. Saeed Ghodsi and Zahra Hosseini extracted the data. Zahra Hosseini and Saeed Ghodsi wrote the draft and revised the manuscript Saeed Ghodsi and Mehrnaz Mohebi performed statistical analysis. Seyed‐Ali Sadre‐Bafghi, Saeed Ghodsi,and Zahra Hosseini prepared the Figures. Seyed‐Ali Sadre‐Bafghi and Saeed Ghodsi presented the tables. All authors read and approved the final manuscript.

## Supporting information


**Figure S1** Subgroup analysis of the association between high Apo‐B and MACE after primary PCI

## Data Availability

Data of this manuscript will not be released unless in limited form after evaluation of an official request.
