# Supplementary material for: Prognostic implications of calculated Apo‐lipoprotein B in patients with ST‐segment elevation myocardial infarction undergoing primary percutaneous coronary intervention: Outcome is tied to lower cut‐points
Source: Clin Cardiol. 2021 May 4;44(6):824–32. doi: 10.1002/clc.23610 (PMC8207970; doi:10.1002/clc.23610)
Supplement: Supplementary file 1 — Figure S1 Subgroup analysis of the association between high Apo‐B and MACE after primary PCI [file CLC-44-824-s001.docx]

**Female**

**Male**

**Age <59**

**Age ≥ 59**

**TG<250**

**TG≥250**

**Non-smoker**

**Ever smoker**

**Nondiabetic**

**Diabetic**

**HTN (-)**

**HTN (+)**

**Normal TIMI**

**Reduced post-PCI TIMI**

**No Opium**

**Current Opium use**

**Former Opium use**

**LAD**

**LCX**

**RCA**

**Graft**

**Ostial-Proximal**

**Mid-distal**

**COPD (-)**

**COPD (+)**

**(-) Cardiogenic shock**

**Cardiogenic shock (+)**

**Prior CABG (-) Prior CABG (+)**

**Prior PCI (-)**

**Prior PCI (+)**

**Non-HDL <100**

**Non-HDL ≥ 100**

**LVEF< 40**

**LVEF≥ 40**

**BMI <25**

**BMI ≥25**

**<25 Lesion Length**

**LL ≥25mm**

**CVA (-)**

**CVA (+)**

**<90 Pain-to-door time**

**PD ≥ 90 min**

**High HDL**

**Low HDL**

**LDL <70**

**LDL ≥70**

**Hemodialysis (-)**

**Hemodialysis (+)**

**Figure S1. Subgroup analysis of the association between high Apo-B and MACE after primary PCI**
